# Impact of emotional intelligence on anxiodepressive disorders in nursing staff in the intensive care unit

**DOI:** 10.1192/j.eurpsy.2024.1229

**Published:** 2024-08-27

**Authors:** M. Kahloul, I. Kacem, A. Ghenim, M. Ajmi, W. Naija, A. Chaouch, N. Mrizak

**Affiliations:** ^1^Anesthesia and Intensive Care Department, Sahloul Academic Hospital; ^2^Occupational Medicine Department, Farhat Hached Academic hospital, Sousse, Tunisia

## Abstract

**Introduction:**

Working in the intensive care unit (ICU) often involves intensely stressful and emotional situations, which can be strong predictors of poor mental health. Healthcare workers are required to perceive, understand, manage, and use their emotions to provide quality care.

**Objectives:**

To evaluate the impact of emotional intelligence (EI) on anxiodepressive disorders in nursing staff in the ICU.

**Methods:**

This was a descriptive, cross-sectional, analytical study conducted among nursing staff in the ICUs of two hospitals in Tunisia. Data were collected over a 3-month period. EI was assessed using the SSEIT self-report test, and the hospital anxiety and depression scale (HADS) was used to measure anxiodepressive disorders. Sociodemographic aspects were also taken into account.

**Results:**

We included 92 healthcare workers. The majority were women (67.4%) with an average age of 25 to 54 years. Nurses represented 58.7% of the study population. About half had less than 5 years of occupational seniority. In terms of lifestyle habits, 76% were smokers, 90.2% did not consume alcohol, and 53% had no leisure activities. The majority had no personal, family, or medical psychiatric history.

The mean EI score was 109.9, ranging from 62 to 150. Anxiety was present in 43.49% of participants and depression in 51.08%.

A significant association was observed between anxiety and the perception of emotions (p=0.0196) and the management of others’ emotions (p=0.0261).

As for depression, a significant association was observed between perception of emotions and depression (p=0.0259), as well as between management of others’ emotions and depression (p=0.0126). EI was positively associated with HADS (p=0.0281), with a correlation value of 0.114.

**Conclusions:**

Caregivers with anxiodepressive disorders had significantly lower levels of EI than those without anxiodepressive disorders, suggesting that EI maybe a protective factor against these disorders

**Disclosure of Interest:**

None Declared

